# The False-Positive Rate of Synovial Fluid Culture at a Single Clinical Laboratory Using Culture Bottles

**DOI:** 10.7759/cureus.55641

**Published:** 2024-03-06

**Authors:** Carl Deirmengian, Krista Toler, Varun Sharma, John L Miamidian, Alex McLaren

**Affiliations:** 1 Orthopaedic Surgery, The Rothman Orthopaedic Institute, Philadelphia, USA; 2 Orthopaedic Surgery, Thomas Jefferson University, Philadelphia, USA; 3 Diagnostics Research and Development, Zimmer Biomet, Warsaw, USA; 4 Medicine, University of Arizona College of Medicine - Phoenix, Phoenix, USA; 5 Diagnostics Research and Development, Zimmer Biomet, Claymont, USA; 6 Orthopaedic Surgery, University of Arizona College of Medicine - Phoenix, Phoenix, USA

**Keywords:** shoulder, hip, knee, laboratory, synovial fluid culture, periprosthetic joint infection, pji

## Abstract

Introduction

Synovial fluid (SF) cultures can yield false-positive or negative results when diagnosing periprosthetic joint infection (PJI). False-positives may arise during sample collection or from laboratory contamination. Understanding false-positive SF culture rates is crucial for interpreting PJI laboratory data, yet clinical laboratories rarely report these rates. This study aimed to define the false-positive SF culture rate at a major specialized clinical laboratory.

Methods

This study retrospectively analyzed prospectively collected data at a single clinical laboratory that receives SF for clinical testing for PJI. A total of 180,317 periprosthetic SF samples from the hip, knee, and shoulder were identified from January 2016 to December 2023, which met the inclusion criteria for this study. Samples were classified by both a modified 2018 International Consensus Meeting (ICM) score and an inflammation score that combined the SF-C-reactive protein, alpha-defensin, SF-white blood cell count, and SF-polymorphonuclear% into one standardized metric. Logistic regression was utilized to evaluate the impact of various collection-based characteristics on culture positivity, including inflammation biomarkers, the source joint, quality control metrics, and days of specimen transport to the laboratory. SF culture false-positivity was calculated based on the ICM category of “not-infected” or low inflammation score.

Results

Overall, 13.3% (23,974/180,317) of the samples were associated with a positive culture result: 12.5% for knee samples, 20.3% for hip samples, and 14.7% for shoulder samples. The false-positive SF culture rate among 131,949 samples classified as “not-infected” by the modified 2018 ICM definition was 0.47% (95%CI: 0.43 to 0.51%). Stratification by joint revealed a false-positive rate of 0.34% (95%CI: 0.31 to 0.38%) for knee samples, 1.24% (95%CI: 1.05 to 1.45%) for hip samples, and 3.02% (95%CI: 2.40 to 3.80%) for shoulder samples, with p < 0.0001 for all comparisons. The false-positive SF culture rate among 90,156 samples, representing half of all samples with the lowest standardized inflammation scores, was 0.47% (95%CI: 0.43 to 0.52%). Stratification by joint revealed a false-positive rate of 0.33% (95%CI: 0.29 to 0.37%) for knee samples, 1.45% (95%CI: 1.19 to 1.77%) for hip samples, and 3.09% (95%CI: 2.41 to 3.95%) for shoulder samples, with p<0.0001 for all comparisons. Multivariate logistic regression demonstrated the joint source (hip, shoulder) and poor sample quality as collection-based factors associated with a false-positive culture. Evaluation of a cohort of samples selected to minimize collection-based causes of false-positive culture demonstrated a false-positive rate of 0.30%, representing the ceiling limit for laboratory-based SF culture false-positivity.

Conclusions

This study utilizes two methods to estimate the false-positive SF culture rate at a single specialized clinical laboratory, demonstrating an overall false-positive rate of approximately 0.5%. Stratification of samples by source joint demonstrated that periprosthetic SF from the shoulder and hip have a substantially higher false-positive culture rate than that of the knee. The lowest false-positive SF culture rate (0.30%) was observed among samples from the knee-passing quality control. Culture positivity due to contamination at this specific laboratory is less than 0.30% because all specimens undergo identical processing.

## Introduction

Synovial fluid (SF) culture is a critical component of the clinical diagnostic tests used to identify joint infections. Historically, the results of cultures were exclusively relied upon to determine the presence of a joint infection [1]. Recent research into periprosthetic joint infections (PJI), however, has revealed the potential for infections to exist despite negative culture results [2]. This phenomenon, known as "culture-negative infection," is recognized across various medical fields, particularly in relation to implant-related infections. Additionally, it is also now well established that a positive culture in the absence of other positive tests for infection may represent false-positive culture results [3-6]. Contemporary authoritative definitions of PJI, such as those suggested by the Musculoskeletal Infection Society (MSIS) and International Consensus Meeting (ICM) definitions, now generally regard a single positive culture as neither a necessary nor sufficient criterion for diagnosing an infection [4,7,8]. The acknowledged occurrence of both false-positive and false-negative culture results has significantly impacted the diagnostic approach to PJI.

False-positive cultures in the diagnosis of PJI can present a particularly challenging scenario for clinicians, requiring careful interpretation and consideration. The occurrence of a false-positive culture introduces a dilemma, potentially leading to unnecessary treatment for an infection that does not exist, or conversely, complicating the clinical picture and delaying appropriate treatment for a patient with an aseptic problem. There are several potential mechanisms leading to false-positive growth in SF cultures. First, collection-based contamination could occur due to bacterial contamination of the materials used for collection or during the procedure for fluid aspiration. Second, laboratory-based contamination could occur if the laboratory environment or processes, including the handling and incubation of cultures, introduce contaminants into the culture medium. These mechanisms collectively contribute to a baseline rate of false-positive cultures from SF at every clinical laboratory. Understanding the rate of culture false positivity in any given laboratory would benefit the clinician interpreting laboratory data to diagnose infection.

Traditionally, the baseline rate of false-positive culture results has not been a metric that has been examined or reported by individual clinical laboratories. Given the importance of understanding the baseline false-positive culture rate when interpreting positive culture results, we examined the culture and biomarker results from SF at one specialized clinical laboratory receiving samples from institutions across the United States. The purpose of this study was to estimate the overall false-positive SF culture rate at one clinical laboratory.

## Materials and methods

Study design and approval

We conducted a retrospective analysis of prospectively collected SF samples deidentified according to the Institutional Review Board-approved protocol [WIRB Copernicus Group (WCG IRB)]. Copy-editing for grammar, spelling, and punctuation was performed using ChatGPT 4.0 without generating content. Cureus is the sole submission site.

Data collection

We analyzed deidentified SF samples submitted to CD Laboratories (Zimmer Biomet, Towson, MD) for a diagnostic PJI workup. Samples originated from standard clinical care across the U.S. and were not sent specifically for this study. Data from laboratory instruments were transferred digitally to the CGM LabDAQ (CompuGroup Medical, Austin, Texas), operating on Microsoft SQL Server.

Laboratory testing

Healthcare providers sent aspirated SF samples in vacutainer tubes with EDTA or without additives (Becton, Dickinson, and Company, Franklin Lakes, NJ) via overnight courier. Fluid cultures were performed using the BacT/Alert system (Biomerieux, Inc.) with both aerobic and anaerobic bottles, adding laked horse blood for shoulder samples. Cultures were incubated for seven days (hip/knee) or 14 days (shoulder) before being considered negative.

SF-CRP and alpha-defensin immunoassays were performed as previously described [9]. SF-WBC and SF-PMN% were determined using an automated cell counter with manual counting for SF-WBC > 3000 cells/µl as previously described [10].

Data filtering for the current study

We filtered the raw database for samples indicating the knee, hip, or shoulder collected between January 1, 2016, and December 31, 2023, for PJI workups. Out of 255,193 samples, 189,575 had requested microbiological cultures. A total of 180,317 samples with complete SF-CRP, alpha-defensin, SF-WBC, SF-PMN%, and SF-culture results were analyzed.

Infection classification and inflammation score

First, to allow for categorical classification as “not infected” using a gold standard, a modified 2018 ICM score [11] was calculated based on the minor preoperative criteria from SF (Table 1).

**Table 1:** Points attributed to synovial fluid laboratory tests as part of the modified 2018 International Consensus Meeting definition of periprosthetic joint infection SF: synovial fluid, CRP: C-reactive protein, AD: alpha-defensin, WBC: white blood cells, PMN%: polymorphonuclear cell percent.

Test	Threshold	Points
SF-CRP positive	4.45 mg/L	2
Alpha-defensin or SF-WBC positive	S/CO=1; 3,000 cells/µl	3
PMN% positive	70%	2
SF-culture	Positive	2

The score used in this study was considered a modified score because the serum CRP (threshold = 10 mg/L) was replaced by SF-CRP (threshold = 4.45 mg/L) for scoring [12]. This decision is supported by robust evidence in the literature demonstrating not only a correlation between the serum and SF-CRP but also a consistent demonstration that the SF-CRP is either diagnostically equivalent or superior to the serum CRP [13,14]. The clinical threshold and points associated with each test were based on the 2018 ICM criteria (Table 1). False-positive SF culture was defined as a positive culture in the setting of a “not-infected” classification (score 0-2).

Second, to eliminate the bias introduced by points attributed to culture in the 2018 ICM criteria, an inflammation score was assigned to each sample, combining the SF-CRP, alpha-defensin, SF-WBC, and SF-PMN%. The metric was developed through a sequence of standard data transformations, including logarithmic transformation, outlier clipping, standardization, and principal component analysis (PCA). Analysis of the distribution of the laboratory tests within the study's sample set revealed significant skewness in the results for SF-CRP, alpha-defensin, and SF-WBC. To address this skewness, logarithmic transformations were applied. Subsequently, outliers were identified and replaced using a standard criterion of three standard deviations above and below the mean, minimizing their impact on test results. Following this, all four tests underwent standardization as part of a PCA, which was executed using GraphPad Prism software, version 9.5.0 (GraphPad Software, Inc., La Jolla, CA). The score derived from the first principal component, accounting for 78.8% of the proportion of variance, was then employed as the inflammation score for each sample in the dataset. This approach provided a continuous metric for the inflammatory state as compared to the categorical scoring system based on the 2018 ICM criteria.

For the purposes of analysis, samples were ranked by inflammation score and then divided into 10 equal groups, or rank-order deciles. The first decile therefore had the samples with the lowest inflammation scores, and the last decile had the samples with the highest inflammation scores. Culture positivity was assessed across all inflammation rank-order deciles to determine the relationship between inflammation and culture positivity.

Logistic regression by sample feature

Logistic regression was performed in order to identify those factors associated with false-positive cultures. Analyzed features included source joint, transport days, inflammation score, and quality control metrics. Quality control metrics followed protocols assessing absorbance at 280 nm and SF-RBC count, categorizing samples as failing if they were outside established ranges. To elucidate the contribution of each feature to the likelihood of a false-positive culture, the coefficients derived from the logistic regression were examined.

Ceiling rate of the false-positive cultures due to laboratory contamination

In the logistic regression analysis, the features used were specific to the SF characteristics of the samples prior to their arrival at the laboratory, which implies they should not influence rates of laboratory-based contamination of SF. For instance, factors such as the source joint, SF quality, and the duration of transport to the laboratory are expected to be unrelated to the contamination occurring within the laboratory environment. This distinction is crucial in differentiating between "collection-based contamination" and "laboratory-based contamination."

To accurately assess the maximal limit of the false-positive rate attributable to laboratory contamination, a sub-cohort of samples was strategically selected, based on logistic regression, to minimize potential sources of false positivity not related to the laboratory processes. This selection criteria included samples with an 2018 ICM classification of "not-infected," inflammation ranking lower than the 50th percentile, being sourced from the knee, and exhibiting good sample quality metrics. This subset, comprising 79,655 samples, represented those with the lowest likelihood of pre-laboratory, or collection-based, contamination leading to false-positive culture results. Utilizing this cohort allowed for the minimization of collection-based contamination, providing an estimation of the maximum potential rate of contamination attributable solely to laboratory processes.

Statistical analysis

The chi-squared test with Yates' correction and linear trend analysis assessed significant differences in proportions and trends. The Wilson score interval calculates 95% confidence intervals for proportions.

## Results

Sample characteristics

A total of 180,317 periprosthetic SF samples were analyzed in this study. Of these, 159,011 samples were obtained from the knee, 18,174 from the hip, and 3,132 from the shoulder. Overall, 13.3% (23,974/180,317) of the samples were associated with a positive culture result. Culture positivity was further stratified by source joint, revealing rates of 12.5% for knee samples, 20.3% for hip samples, and 14.7% for shoulder samples.

False-positive culture rate using modified 2018 ICM classification

**Figure 1:**
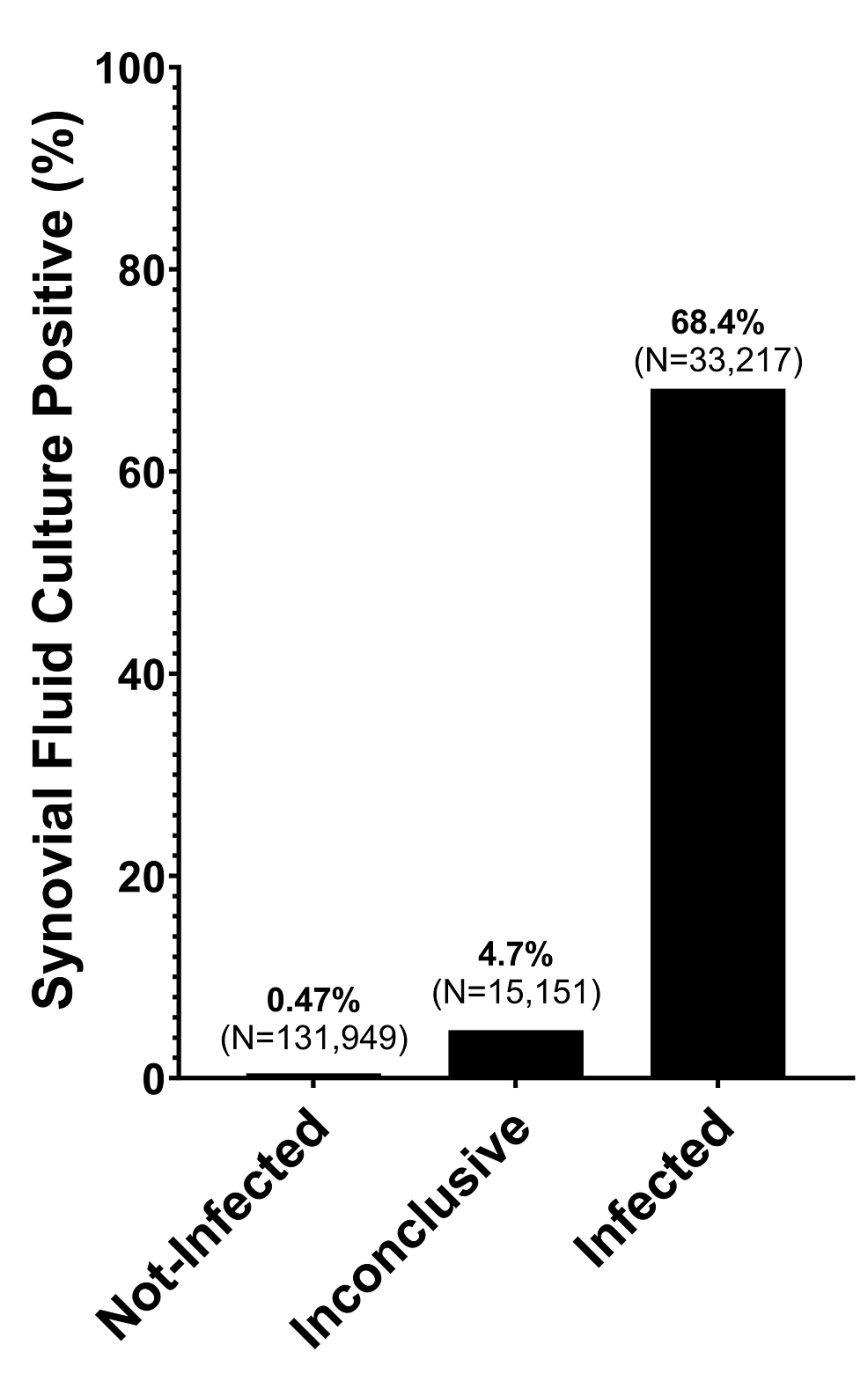
Culture positivity by modified 2018 International Consensus Meeting definition

Culture positivity was calculated for categories within the modified 2018 ICM framework, as illustrated in Figure 1. Samples with a score of 0 or 2, categorized as “not-infected” (n=131,949), had a false-positive culture rate of 0.47% (95% CI: 0.43 to 0.51%). Stratification by joint source revealed a false-positive rate of 0.34% (95% CI: 0.31 to 0.38%) for knee samples, 1.24% (95% CI: 1.05 to 1.45%) for hip samples, and 3.02% (95% CI: 2.40 to 3.80%) for shoulder samples categorized as "not-infected." A comparative analysis of the false-positive culture rates across these different joint sources (hip, knee, and shoulder) indicated significant differences, with all comparisons yielding a p-value of less than 0.0001. Analysis of shoulder samples at seven days of incubation revealed a false-positive SF culture rate of 1.90% (95% CI: 1.42 to 2.54%), revealing that 37.1% of false-positive samples from the shoulder occurred during the extended culture incubation period.

False-positive culture rate using an inflammation score

**Figure 2:**
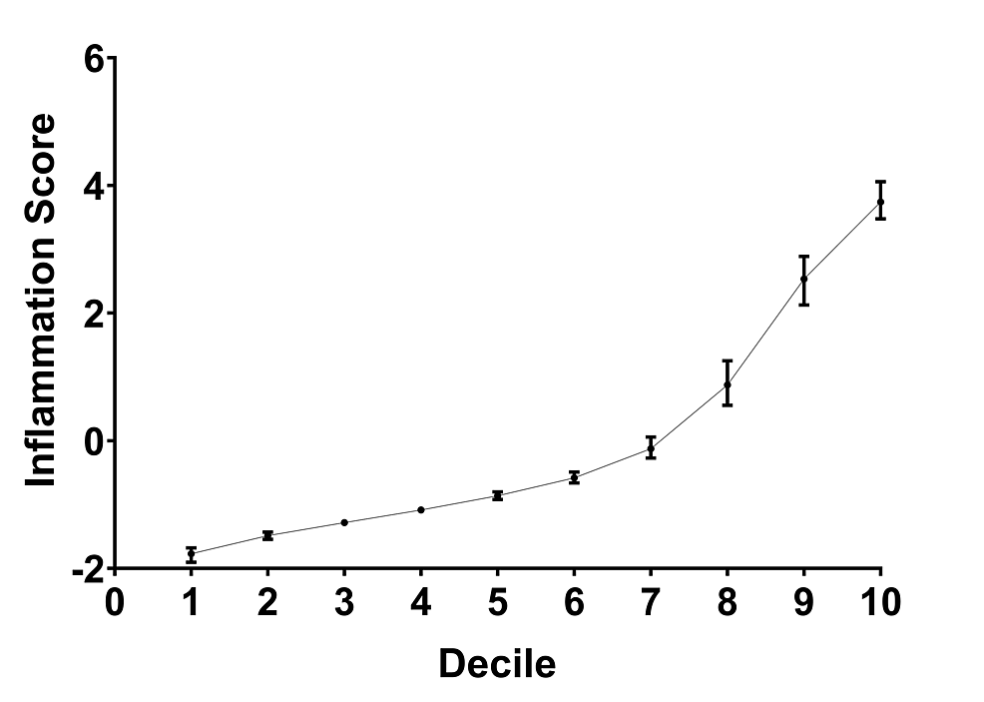
Inflammation scores across study samples All study samples were divided into equal frequency deciles by inflammation score rank-order. The median inflammation score for each decile group is shown on the Y-axis, with interquartile ranges.

**Figure 3:**
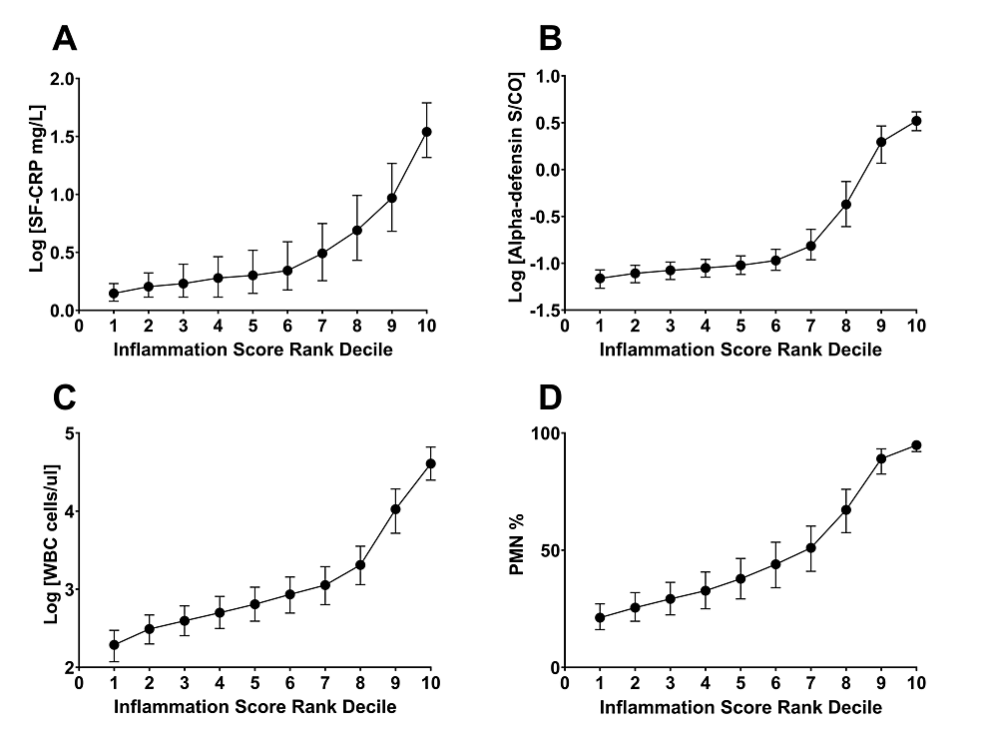
Biomarker values across study samples Panel A depicts the median with interquartile intervals for SF-CRP. Panel B depicts the median with interquartile intervals for AD. Panel C depicts the median with interquartile intervals for SF-WBC. Panel D depicts the median with interquartile intervals for SF-PMN%. All samples are grouped by inflammation score rank-order decile. When graphing SF-CRP, the log[SF-CRP+1] was calculated to avoid errors when calculating log(0). Logarithmic graphs of the SF-CRP, AD, and SF-WBC are depicted to allow for proper visualization of scale. SF: synovial fluid, CRP: C-reactive protein, AD: alpha-defensin, WBC: white blood cells, PMN%: polymorphonuclear cell percent.

An inflammation score, a composite metric of SF-CRP, alpha-defensin, SF-WBC, and SF-PMN%, was assigned to each sample. The samples were divided into 10 rank-order deciles based on increasing inflammation scores, with each decile containing an equal number of samples (Table 2). As expected based on the methodology, median inflammation scores increased with each successive decile (Figure 2). Similarly, the median values for each biomarker rose correspondingly (Figure 3).

**Table 2:** Characteristics of all samples by rank-order decile All study samples were ordered by inflammation score and divided into ten rank-order deciles of equal frequency. Decile grouping characteristics are shown. The proportion of culture-positive samples can be observed to increase in deciles six through ten, where the synovial fluid inflammation rises to a level typical of infected samples.

Rank-order decile	Inflammatory score		Decile (n)	Culture-positive sample number	Culture-negative sample number	Proportion culture positive	95% confidence interval	
	Lower edge	Upper edge					Lower	Upper
1	−3.66	−1.61	18,031	88	17,943	0.49%	0.40%	0.60%
2	−1.61	−1.38	18,031	76	17,955	0.42%	0.34%	0.53%
3	−1.38	−1.18	18,031	79	17,952	0.44%	0.35%	0.55%
4	−1.18	−0.98	18,032	88	17,944	0.49%	0.40%	0.60%
5	−0.98	−0.73	18,031	96	17,935	0.53%	0.44%	0.65%
6	−0.73	−0.39	18,033	110	17,923	0.61%	0.51%	0.73%
7	−0.39	0.28	18,032	247	17,785	1.37%	1.21%	1.55%
8	0.28	1.69	18,032	1038	16,994	5.76%	5.43%	6.11%
9	1.69	3.21	18,033	7638	10,395	42.36%	41.64%	43.08%
10	3.21	5.15	18,031	14,514	3517	80.49%	79.91%	81.07%

**Figure 4:**
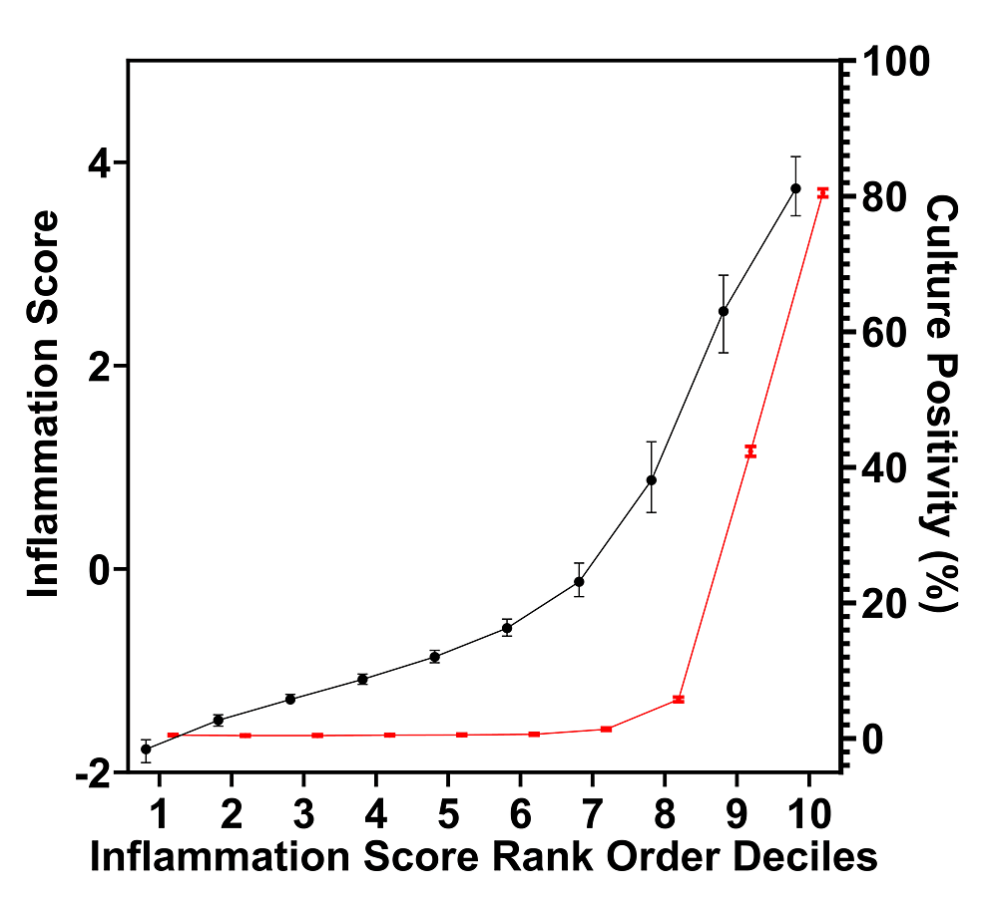
Inflammation score and synovial fluid culture positivity The median inflammation score (black line) and percent culture positivity (red line) are depicted for each sample rank-order inflammation score decile 1–10. Deciles 1–5 demonstrate consistently low baseline rates of culture positivity despite increasing inflammation, whereas deciles 6–10 reveal rapidly increasing culture positivity with higher inflammation scores.

A calculation of culture positivity by rank-order decile demonstrates low levels of culture positivity with lower inflammation scores and rapidly increasing culture positivity at higher inflammation scores (Figure 4). The five lowest deciles, half of all samples (n = 90, 156), demonstrated low rates of culture positivity ranging from 0.42% to 0.53%. Chi-squared testing for a linear trend of culture positivity among these five lowest deciles demonstrated no statistically significant linear trend. This indicated a false-positive culture rate of 0.47% (95% CI: 0.43 to 0.52%), averaged across 90,156 samples (deciles 1-5) with the lowest inflammation scores.

An equivalent analysis to determine the baseline culture positivity across the lowest 5 inflammation score rank order deciles was conducted after stratifying the inflammation score by joint source. This stratification revealed baseline false-positive culture rates (mean of first 5 deciles) for samples aspirated from different joints: 0.33% (95% CI: 0.29 to 0.37%) for knee samples, 1.45% (95% CI: 1.19 to 1.77%) for hip samples, and 3.09% (95% CI: 2.41 to 3.95%) for shoulder samples, with p < 0.0001 for all comparisons.

Logistic regression to minimize collection-based causes of false-positive SF culture

A multivariate logistic regression analysis was conducted among samples ranking in the first five deciles of the inflammation score to ascertain the associations between collection-based features of the aspirate and culture false-positivity. Analysis of beta parameter estimates demonstrated that the joint source (hip and shoulder) and the quality control determination (poor) of the sample were associated with an increase in SF culture false-positivity (Table 3). Through the first five rank-order deciles, the inflammation score and number of transport days of SF to the laboratory had no association with false-positive culture results.

**Table 3:** Multivariate logistic analysis results factors associated with false-positive culture Multiple logistic regression was performed on samples in the first 5 inflammation score rank-order deciles, where no increase in culture positivity is observed with increasing inflammation. Standard coefficients and p-values for synovial fluid factors are listed. Shoulder and hip samples were associated with the highest weighting as factors associated with false-positive synovial fluid culture results. Poor sample quality, based on red blood cell count and absorbance readings, was also associated with the likelihood of false-positive cultures.

Parameter estimates	Variable	Estimate	Standard error	Lower 95%CI	Upper 95%CI	P-value
β0	Intercept	−5.53	0.19	−5.89	−5.17	<0.0001
β1	Joint_Type[Hip]	1.26	0.13	0.99	1.51	<0.0001
β2	Joint_Type[Shoulder]	2.20	0.16	1.89	2.49	<0.0001
β3	Inflammation score	−0.17	0.14	−0.44	0.10	0.2231
β4	Transport days	0.00	0.00	−0.01	0.01	0.4681
β5	Sample quality	0.77	0.14	0.48	1.05	<0.0001

A cohort of samples was selected to minimize collection-based sources of false positivity based on the regression analysis results. In order to minimize the likelihood of true positive cultures, samples in this cohort not only had to be classified as “not-infected” by the modified 2018 ICM criteria but also had to rank in the lower 5 inflammation score deciles. To minimize collection-based false positivity, samples from the knee with a good quality assessment were included based on the result of multiple logistic regression. Of the samples in this cohort, only 244 of the 79,655 samples, amounting to 0.30% (95% CI: 0.27-0.34%), yielded a positive SF culture.

## Discussion

In this retrospective analysis of 180,317 periprosthetic SF samples, we established benchmarks for false-positive SF culture rates at a specialized clinical laboratory. Utilizing the modified 2018 ICM score and a biomarker-based inflammation score, we determined false-positive rates. The modified 2018 ICM score indicated a 0.47% false-positive rate among "not-infected" samples, and the inflammation score method also showed a 0.47% rate. We observed clear disparities in false-positive rates by aspiration source joint, with hip and shoulder aspirates being more prone than knees. A ceiling of 0.30% was identified for the false-positive culture rate due to laboratory-based contamination. To the best of our knowledge, this study is the first to estimate false-positive SF culture rates using extensive data from a single specialized laboratory.

Our study employed two main methods to determine the false-positive rate of SF cultures in a large laboratory setting. We first applied a modified 2018 ICM criteria [4], scoring each sample and finding a 0.47% false-positive rate. To eliminate the binary limitations of the ICM system and bias from including cultures in scoring, we also used a continuous metric: a combined inflammation score from four SF biomarkers. This analysis also showed a 0.47% culture positivity rate among samples with low inflammation scores, representing the baseline rate when minimizing inflammation. Together, these methods highlight a key finding: culture positivity in a large set of low-inflammation samples is about 0.5%, providing an initial estimate of the false-positive rate from both collection and laboratory sources.

Following the establishment of this baseline rate of culture positivity, we further analyzed the data to determine the ceiling rate of false-positive cultures due to laboratory-based contamination. A key assumption of our analysis is that laboratory contamination occurs independently of the collection characteristics. For instance, there is no inherent rationale to suspect that a sample from the hip is more prone to contamination during handling in the laboratory than one from the knee. Guided by this assumption, we employed logistic regression to reveal the collection-based sample attributes most strongly associated with positive cultures in cases of minimal inflammation. We discovered that samples from the shoulder and hip joints were more prone to false-positive cultures. This finding might reflect the higher presence of commensal skin organisms [15,16] and/or the diversity in aspiration techniques used by various clinical practitioners [17,18] at these anatomic sites. Additionally, samples failing quality control assessments, including those with excessive red blood cell counts or those with absorbance readings resembling saline or a contrast agent more than SF, were associated with an elevated rate of false-positive cultures. By isolating a subset of SF samples that excluded these identified collection-based factors, we found that the remaining low-inflammation knee joint samples, which passed quality control, manifested a low false-positive rate of 0.30%. Consequently, this rate signifies the ceiling false-positive rate attributable to laboratory sources. It is plausible, however, that the actual rate could be lower, as some of these positives might be due to other collection-based contamination or true positive cultures. Most importantly, our data suggest that efforts focused on reducing the collection-based contamination rate for shoulder and hip arthroplasty aspirations may be good targets to successfully lower false-positive SF culture rates.

The overall false-positive SF culture rate of approximately 0.5% identified in this study contrasts with the few studies reporting false-positive rates in the literature. The false-positive SF culture rate calculated in this study is 5 to 10 times lower than that typically described in modern literature. Bingham et al. [3] found a false-positive culture rate of 12% when using the Musculoskeletal Infection Society Criteria as a gold standard. Ryu et al. [19], in their evaluation of polymerase chain reaction detection of PJI, found an SF culture false-positive rate of 3.6% (1/28) among patients with aseptic arthroplasty failure. Melendez et al. [20] found an SF culture positivity of 2.6% among 196 samples from patients with aseptic failure. A recent study by Ji et al. [21] demonstrated a false-positive SF culture rate of 7.1% among patients deemed aseptic. Obviously, the false-positive rate determined in any specific study will depend on many factors, including the definition of PJI, the clinical laboratory processing the samples, and the institution’s aspiration techniques. The current study presents data from institutions across the nation with a large number of samples for analysis from one clinical laboratory. Therefore, this study likely provides a more accurate nationwide representation of collection-based contamination and a conservative estimate of the laboratory-based false-positive rate for one clinical laboratory, suggesting much lower false-positive SF culture rates can be achieved than what has been previously described.

While our study provides findings on false-positive rates in SF cultures, it is important to consider its limitations. First, the study's results are from a single clinical laboratory that specializes in SF testing, which raises questions about their applicability to other laboratories with different protocols or techniques. This may limit the extrapolation of our findings to broader clinical contexts. Furthermore, the exclusive use of the blood culture bottle method in this laboratory limits the universality of our results. Different methodologies or technological approaches in SF culture may yield varying false-positive rates, highlighting the necessity for more detailed methodological studies in this field. Additionally, the retrospective design of our research introduces potential biases and unmeasured confounding factors, which could influence the observed outcomes.

## Conclusions

This retrospective study analyzed 180,317 SF samples from a specialized clinical laboratory to determine the false-positive culture rate in diagnosing PJI. Using two methods to determine false-positive rates, this study found an overall SF culture false-positive rate of 0.47%.

Logistic regression was used to identify several factors contributing to collection-based false-positive cultures, including samples from the hip and shoulder and samples failing to pass quality control based on red blood counts and absorbance. When minimizing collection-based causes of culture false positivity, the residual rate of unexpected positive SF culture in the setting of knee samples passing quality control was 0.30%, representing a ceiling rate for laboratory-based contamination of samples.
